# Clinical management of intestinal malrotation in different age groups

**DOI:** 10.1007/s00383-024-05796-9

**Published:** 2024-07-20

**Authors:** Süleyman Arif Bostancı, Can İhsan Öztorun, Elif Emel Erten, Fahri Akkaya, İrem Akbaş, Vildan Selin Çayhan, Aslı Nur Abay, Sabri Demir, Ahmet Ertürk, Müjdem Nur Azılı, Emrah Şenel

**Affiliations:** 1 Department of Pediatric Surgery, Ankara Bilkent City Hospital, Childrens’ Hospital, Universiteler Boulvard, 1604. Street, Çankaya, 06800 Ankara, Turkey; 2 Faculty of Medicine, Department of Pediatric Surgery, Ankara Yildirim Beyazit University, Ankara, Turkey; 3 Department of Pediatric Surgery, Health Science University, Ankara Bilkent City Hospital, Childrens’ Hospital, Ankara, Turkey

**Keywords:** Malrotation, Volvulus, Vomiting, Children

## Abstract

**Purpose:**

Intestinal malrotation, characterized by abnormal intestinal positioning, can lead to severe complications like volvulus and internal hernias, especially in neonates and children. Our aim was to evaluate the diagnostic methods, treatment results and postoperative follow-up of pediatric patients treated for intestinal malrotation.

**Methods:**

This retrospective study reviewed medical records of pediatric patients who underwent surgery for intestinal malrotation between January 2013 and January 2022. Data on demographics, symptoms, diagnostic approaches, surgical interventions, and postoperative outcomes were analyzed.

**Results:**

The study included 45 patients, with a male predominance (68.8%). Ages ranged from 1 day to 15 years, averaging 1.54 years. Presenting symptoms were acute abdomen (*n* = 21) and chronic abdominal pain with vomiting (*n* = 24). Diagnoses were established via physical exams and imaging, including upper gastrointestinal contrast studies and abdominal ultrasonography. All patients received the Ladd procedure, with some requiring necrotic bowel resection due to volvulus.

**Conclusion:**

The diagnosis and management of pediatric intestinal malrotation present significant challenges due to its variable symptoms and potential for life-threatening complications. Early and accurate diagnosis, followed by appropriate surgical management, is crucial. This study emphasizes the importance of diligent postoperative follow-up to identify and mitigate complications, particularly in younger and severely affected patients.

## Introduction

Intestinal malrotation, known as bowel rotation anomaly, is a common cause of intestinal obstruction in neonates and can lead to life-threatening complications such as internal hernias and midgut volvulus. Approximately 50% of affected cases present symptoms during the neonatal period [1]. The condition arises from errors in intestinal fixation and the formation of a narrow mesenteric base during fetal development [2–4]. Malrotation is reported to occur in one out of every 500 births, with symptomatic cases occurring once in 6000 births. Notably, neonates represent 50% of these patients, while children under one year account for 75% [5, 6]. Symptoms in neonates and infants typically include acute midgut volvulus along with Ladd's bands, whereas older children may present with chronic abdominal pain and bilious vomiting [7, 8].

The aim of this study is to present the demographic data of patients operated on for malrotation, to compare the diagnosis and treatment outcomes of cases presenting with acute abdominal symptoms versus chronic abdominal pain and vomiting, and to evaluate the results of postoperative follow-up.

## Materials and methods

Ethical approval for this retrospective study was obtained from our hospital's Clinical Research Ethics Committee (E2-23-3830). We reviewed the medical records of patients diagnosed with isolated intestinal malrotation and operated on at our clinic from January 2013 to January 2022. Collected data included patient demographics, presenting symptoms, diagnostic assessments, intraoperative findings, time to full enteral feeding, length of hospital stay, and postoperative complications.

Patients were categorized for either elective or emergency surgery based on their condition. Patients presenting with an acute abdomen and diagnosed with volvulus underwent emergency surgery. For those presenting with chronic abdominal pain and vomiting, malrotation was initially suspected, and diagnostic evaluation typically began with an abdominal Doppler ultrasound to assess the relationship between the SMA and SMV, followed by an elective operation based on a stomach-duodenum X-ray**.**

All surgical interventions for intestinal malrotation in this study were performed adhering to the Ladd’s procedure, which is the standard treatment for this condition. The procedure involves the counterclockwise detorsion of the volvulus, if present, division and excision of Ladd’s bands, widening of the mesenteric base to prevent future volvulus, and placement of the small intestines on the right and the colon on the left side of the abdomen. For neonates and young infants, where urgency was paramount due to acute presentation, these steps were swiftly executed to minimize time under anesthesia and reduce complications. In older children, where diagnostic findings were less acute, the procedure allowed for careful examination of the entire bowel and assessment for secondary complications such as necrosis or perforation. Specific variations in the procedure, such as the extent of bowel resection required, were based on the intraoperative findings which included the presence and severity of necrotic bowel segments. The decision to resect involved assessing the viability of the bowel using visual inspection.

## Statistical analysis

SPSS 25 (SPSS Inc., Chicago, IL, USA) was used for statistical analyses. Data were analysed in terms of demographic information, clinical findings, diagnostic methods, treatment results and postoperative complications. Categorical variables were expressed as frequencies and percentages, and continuous variables were expressed as means and standard deviations. Differences between groups were analysed using chi-square test and Fisher’s exact test. The relationships between volvulus rates and postoperative complications were analysed using logistic regression analysis. Statistical significance level was determined as *p* < 0.05.

To enhance the accuracy of our statistical representation and address the variability within our data, we have included both the mean and the median values in our analysis. The use of median values is particularly emphasized for variables with non-normal distributions or where outliers are present, such as age, length of hospital stay, and size of resected bowel segments. This approach ensures that our statistical descriptions more faithfully represent the central tendencies of our diverse patient population. Updated tables now reflect these changes, with both mean and median values provided, allowing for a nuanced interpretation of the data.

## Results

The study involved 45 patients, comprising 31 males (68.8%) and 14 females (31.2%), with ages ranging from one day to 15 years, averaging at 1.54 years. The presenting complaints were acute abdomen (*n* = 21) and chronic abdominal pain and vomiting (*n* = 24). All patients presenting with acute abdomen were diagnosed through physical examination. The abdominal X-ray was performed on all patients to rule out intestinal perforation. Of the patients presenting with chronic abdominal pain and vomiting, 15 (62.5%) were diagnosed using Upper Gastrointestinal (UGI) contrast studies and 9 (37.5%) with abdominal ultrasonography. All patients underwent laparotomy, and the Ladd procedure was performed. There were nine patients who underwent necrotic bowel resection due to volvulus. The mean resected bowel length was 72.8 ± 12.5 cm and the mean remaining bowel length was 45 ± 4.6 cm.

Patients were categorized into two groups: under 1 year old (Group 1, *n* = 36) and over 1 year old (Group 2, *n* = 9). Patient’s data according to age groups are shown in Table 1.

**Table 1:** **Demographic data by age groups**

	Group 1 < 1 y.o (*n* = 36)	Group 2 > 1 y.o (*n* = 9)	*p*
Gender			
Female	12	2	0.52
Male	24	7	
Admission complaint to hospital			
Volvulus/acute abdomen	18	3	0.87
Choronic abdominal pain/ vomiting	18	6	
Diagnosis support			
Physical examination	18	2	**0.005**
Upper gastrointestinal (UGI) contrast study	10	5	
Ultrasonography	8	1	
Intraoperative diagnosis			
Only malrotation	16	8	**0.04**
Malrotation and volvulus	20	1	
Postoperative complications			
Anastomotic leakage	3		N/A
Ileus	3		
Sepsis	7		
Short bowel sydrome	3		N/A
Growth retardation	6		N/A
Mortality	7		N/A

No significant difference was observed between the groups in terms of gender and presenting complaints. Diagnoses in the under 1 year old group were most made based on clinical findings, while in the over 1 year old group, they were most commonly made based on UGI contrast studies (*p* = 0.0005).

Intraoperative volvulus was observed in 16 patients (44.4%) in the under 1 year old group, whereas it was observed in one patient (11.1%) in the over 1 year old group (*p* = 0.004).

Postoperative complications, short bowel syndrome, and mortality were only seen in the group 1.

Patients were grouped according to their gender to compare demographic data and clinical findings (Table 2).

**Table 2:** **Differences by gender**

	Female (*n* = 14)	Male (*n* = 31)	*p*
Age (mean(median)(std))	2.06 (0.04) (5.01)	1.31 (0.17) (2.52)	0.25
Weight (mean(median)(std))	7.06 (3.1) (12)	6.38 (3.7) (6.78)	0.53
Admission complaint to hospital			
Volvulus/acute abdomen	6	15	0.165
Choronic abdominal pain/vomiting	8	16	
Intraoperative diagnosis			
Only malrotation	6	18	**0.025**
Malrotation and volvulus	8	13	
Short Bowel Sydrome	0	3	N/A
Growth retardation	0	6	N/A
Mortality	3	4	0.465

No statistical differences were observed between gender groups in terms of age, weight, presenting complaints, and mortality, although volvulus was statistically more frequent in girls. Only male children were observed to have short bowel syndrome and growth retardation during postoperative follow-up.

Twenty-one patients with a preliminary diagnosis of volvulus underwent emergency surgery, while 24 patients with chronic abdominal pain and vomiting were operated on electively with a diagnosis of malrotation. Patients were grouped according to whether they had volvulus to compare demographic data, postoperative complications, and follow-up results, as shown in Table 3.

**Table 3:** **Differences between intraoperative diagnosis**

	Only malrotation (*n* = 24)	Malrotation and volvulus (*n* = 21)	*p*
Gender			
Female	6	8	**0.001**
Male	18	13	
Age (median) (year)	3.48	0.6	**0.01**
Weight (mean ± Std. Dev.)	8.88 ± 10.92	3.97 ± 4.49	0.1
Postoperative complications			
Anastomosis leakage	1	2	**0.02**
Ileus	1	2	**0.02**
Sepsis	1	2	**0.02**
Oral time (mean ± Std. Dev.) (days)	5.73 ± 2.89	6.43 ± 4.31	0.85
Lenght of hospitalization (mean ± Std. Dev.) (days)	15.6 ± 9.5	14.8 ± 12.4	0.33
Short bowel sydrome	1	2	**0.02**
Growth retardation	4	2	0.37
Mortality	1	6	**0.001**

Patients with volvulus had a lower average age and weight but experienced higher rates of postoperative complications (sepsis, brid ileus, anastomotic leak), short bowel syndrome development, and mortality compared to those with malrotation alone (*p* < 0.05). It was also observed that volvulus occurred more frequently in female children compared to male children (*p* < 0.05).

The patients were grouped as survey and mortal to analyze the data based on gender, postoperative complications, and the presence of short bowel syndrome (Table 4). Statistical analysis showed that mortality was higher in patients with volvulus and short bowel syndrome (*p* < 0.05). This analysis indicated that volvulus and short bowel syndrome significantly influenced mortality rates.

**Table 4:** **Results from patients grouped as survey and mortality**

	Survey (*n* = 38)	Mortality (*n* = 7)	*p*
Gender			
Female	11	3	0.46
Male	27	4	
Intraoperative diagnosis			
Only malrotation	23	1	**0.001**
Malrotation and volvulus	15	6	
Postoperative complications			
Anastomosis leakage	3	0	N/A
Ileus	3	0	
Sepsis	0	7	
Short bowel sydrome	1	2	**0.02**

## Discussion

Malrotation is typically diagnosed in the neonatal period, with up to 75% of symptomatic cases occurring in newborns, and nearly 90% within the first year of life [9]. Although symptomatic malrotation beyond infancy is rare, our study found that 80% of cases presented after the age of 1 year, indicating that intestinal malrotation should be considered a potential diagnosis across all age groups. This is supported by a Kids’ Inpatient Database study, which identified malrotation in patients aged 1–18 years, with the majority presenting in their first or second year of life [10].

The classic symptom of bilious vomiting must prompt immediate investigation. However, in our study, most adult patients presented with atypical symptoms that were not initially indicative of intestinal malrotation, aligning with findings from other studies [11, 12].

In the study, we provide a detailed overview of the clinical symptoms and physical examination findings observed in the patients at presentation. The most common symptoms reported included acute abdominal pain, bilious vomiting, and signs of intestinal obstruction in neonates, which were critical in prompting urgent diagnostic evaluations. Physical examinations often revealed signs of abdominal distension and tenderness, which were used to gauge the severity of the condition and to prioritize immediate care. In contrast, older children typically presented with less acute symptoms, such as intermittent abdominal pain or vomiting. These were investigated through a more stepwise diagnostic approach. Detailed documentation of these initial clinical findings was instrumental in guiding subsequent decisions regarding surgical or conservative management. This emphasizes the pivotal role of initial clinical assessment in managing intestinal malrotation [5].

UGI contrast studies are deemed the gold standard for diagnosing malrotation, even in children older than 1 year [13]. In our cohort, 62.5% of patients presenting with chronic abdominal pain diagnosed by UGI and 37.5% diagnosed by abdominal ultrasonography. This high reliance on UGI highlights its importance in diagnosing this condition, where it can distinctly identify malrotation anomalies by the non-crossing of the duodenal loop across the midline or by showing signs of obstruction [13].

Many studies emphasize that most cases of malrotation and volvulus occur acutely within the first month of life [9, 14]. This corroborates our findings in the under- 1-year group, where volvulus incidence was 44.4%, primarily diagnosed through clinical observations. However, the rate of volvulus decreased to 11.1% in the over-1-year group, reflecting typically chronic and vague symptoms, which could contribute to diagnostic delays in older children and adults.

The management of malrotation cases requires the prompt diagnosis and intervention of the condition. In instances where volvulus is present, surgical intervention is required without delay to prevent intestinal ischemia. Anand et al. emphasize the urgency, the variable status of clinical presentations of malrotation in different age groups and the variable treatment approaches applied. It is evident from studies that presentations of malrotation can significantly vary, which may result in delays in diagnosis [11].

Our study also found a higher incidence of volvulus in female children, an observation not commonly reported in the literature [15]. This suggests that gender may influence the development of volvulus, though further research is needed to explore this association.

Research studies that focus on the long-term morbidity of children who have undergone a Ladd procedure highlight the necessity of addressing post-malrotation complications, especially in the context of functional bowel disorders [16]. The findings of our study indicate that patients with volvulus are more susceptible to developing short bowel syndrome and growth retardation, particularly in boys.

Adhesive bowel obstruction following surgery for malrotation has been noted in other studies, with incidences up to 14.2% of cases leading to additional surgeries [17–19]. Our findings were similar, with a significant number of patients returning for surgery due to persistent symptoms or complications like adhesive small bowel obstruction.

Postoperative follow-up in our study revealed that the occurrence of complications, such as short bowel syndrome and developmental delays, was significantly higher in males who underwent surgery compared to females. This aligns with the established body of literature that emphasizes the importance of prompt surgical intervention and accurate early diagnosis in preventing the onset of severe complications, such as bowel necrosis, which are frequently associated with malrotation and volvulus [14]. Furthermore, our findings suggest a significant prevalence of postoperative complications, including short bowel syndrome and sepsis, which are particularly evident in children under the age of one year. These observations highlight the heightened vulnerability of this age group to adverse outcomes associated with volvulus.

The observed mortality rate of 15.5% in this study, while notably high, is reflective of several critical factors that warrant discussion. First, the severity and rapid progression of complications associated with malrotation, such as midgut volvulus, often result in irreversible intestinal damage and sepsis by the time of surgical intervention, particularly in neonates and very young infants. Second, a significant proportion of the mortalities were among patients who presented late to medical attention, where the window for effective intervention was missed. Furthermore, most of these patients presented with additional comorbidities, which further compromised their resilience to adverse outcomes. It is also essential to acknowledge that our centre serves as a referral hub for complex paediatric cases, which may influence the demographic profile of our patient population towards more severe presentations. This discussion is aligning with studies in the literature reporting similar mortality rates in high-risk pediatric populations with bowel malrotation [20, 21]. Our findings emphasize the need for heightened awareness and prompt diagnostic measures in the management of this potentially lethal condition. Further research into early detection and the refinement of surgical techniques is crucial to improve survival rates in this patient group.

Future research should aim to refine diagnostic criteria and explore the potential benefits of integrating newer imaging technologies into the standard diagnostic pathway for suspected malrotation. Additionally, prospective studies are needed to better understand the long-term quality of life and developmental outcomes for these patients, particularly those who undergo surgery as neonates or infants.

## Conclusion

Intestinal malrotation is a complex condition with a broad range of presenting symptoms, from acute to chronic, affecting individuals of all age groups. This study highlights the importance of a prompt and thorough evaluation of bilious vomiting and chronic gastrointestinal symptoms in children. An UGI examination is recommended in all suspected cases to ensure an accurate diagnosis and appropriate surgical intervention. Postoperative follow-up is crucial for the management of long-term complications.

## Data Availability

No datasets were generated or analysed during the current study.

## References

[1] Durkin ET et al (2008) Age-related differences in diagnosis and morbidity of intestinal malrotation. J Am Coll Surg 206(4):658–663. 10.1016/j.jamcollsurg.2007.11.020 18387471 10.1016/j.jamcollsurg.2007.11.020

[2] Millar AJ, Rode H, Cywes S (2003) Malrotation and volvulus in infancy and childhood. Semin Pediatr Surg 12(4):229–236. 10.1053/j.sempedsurg.2003.08.003 14655161 10.1053/j.sempedsurg.2003.08.003

[3] Yousefzadeh DK (2009) The position of the duodenojejunal junction: the wrong horse to bet on in diagnosing or excluding malrotation. Pediatr Radiol 39(Suppl 2):S172–S177. 10.1007/s00247-008-1116-2 19308381 10.1007/s00247-008-1116-2

[4] Zellos A et al (2012) Malrotation of the intestine and chronic volvulus as a cause of protein-losing enteropathy in infancy. Pediatrics 129(2):e515–e518. 10.1542/peds.2011-0937 22271689 10.1542/peds.2011-0937

[5] McVay MR et al (2007) The changing spectrum of intestinal malrotation: diagnosis and management. Am J Surg 194(6):712–717. 10.1016/j.amjsurg.2007.08.035 18005759 10.1016/j.amjsurg.2007.08.035

[6] Torres AM, Ziegler MM (1993) Malrotation of the intestine. World J Surg 17(3):326–331. 10.1007/BF01658699 8337878 10.1007/BF01658699

[7] Dekonenko C et al (2019) The identification and treatment of intestinal malrotation in older children. Pediatr Surg Int 35(6):665–671. 10.1007/s00383-019-04454-9 30810798 10.1007/s00383-019-04454-9

[8] Do WS et al (2019) Predictors of bowel resection during nonelective ladd procedure for pediatric malrotation. J Surg Res 243:419–426. 10.1016/j.jss.2019.05.052 31279268 10.1016/j.jss.2019.05.052

[9] Palanivelu C et al (2007) Intestinal malrotation with midgut volvulus presenting as acute abdomen in children: value of diagnostic and therapeutic laparoscopy. J Laparoendosc Adv Surg Tech 17(4):490–492. 10.1089/lap.2006.0103 10.1089/lap.2006.0103 17705733

[10] Malek MM, Burd RS (2005) Surgical treatment of malrotation after infancy: a population-based study. J Pediatr Surg 40(1):285–289. 10.1016/j.jpedsurg.2004.09.028 15868599 10.1016/j.jpedsurg.2004.09.028

[11] Anand U et al (2018) Comparative study of intestinal malrotation in infant, children, and adult in a tertiary care center in India. Indian J Gastroenterol 37(6):545–549. 10.1007/s12664-018-0914-1 30535747 10.1007/s12664-018-0914-1

[12] Nehra D, Goldstein AM (2011) Intestinal malrotation: varied clinical presentation from infancy through adulthood. Surgery 149(3):386–393. 10.1016/j.surg.2010.07.004 20719352 10.1016/j.surg.2010.07.004

[13] Applegate KE, Anderson JM, Klatte EC (2006) Intestinal malrotation in children: a problem-solving approach to the upper gastrointestinal series. Radiographics 26(5):1485–1500. 10.1148/rg.265055167 16973777 10.1148/rg.265055167

[14] Shalaby MS, Kuti K, Walker G (2013) Intestinal malrotation and volvulus in infants and children. BMJ 347:f6949. 10.1136/bmj.f6949 24285798 10.1136/bmj.f6949

[15] Graziano K et al (2015) Asymptomatic malrotation: diagnosis and surgical management: an American pediatric surgical association outcomes and evidence based practice committee systematic review. J Pediatr Surg 50(10):1783–1790. 10.1016/j.jpedsurg.2015.06.019 26205079 10.1016/j.jpedsurg.2015.06.019

[16] Salo M (2019) Is there a need for bowel management after surgery for isolated intestinal malrotation in children? Pediatr Gastroenterol Hepatol Nutr 22(5):447–452. 10.5223/pghn.2019.22.5.447 31555569 10.5223/pghn.2019.22.5.447 PMC6751105

[17] Miyano G et al (2015) Laparoscopic repair of malrotation: what are the indications in neonates and children? J Laparoendosc Adv Surg Tech A 25(2):155–158. 10.1089/lap.2014.0236 25647302 10.1089/lap.2014.0236

[18] Ooms N et al (2016) Laparoscopic treatment of intestinal malrotation in children. Eur J Pediatr Surg 26(4):376–381. 10.1055/s-0035-1554914 26086418 10.1055/s-0035-1554914

[19] Scalabre A et al (2020) Outcomes of laparoscopic and open surgical treatment of intestinal malrotation in children. J Pediatr Surg 55(12):2777–2782. 10.1016/j.jpedsurg.2020.08.014 32972740 10.1016/j.jpedsurg.2020.08.014

[20] Purcell LN et al (2020) Characteristics of intestinal volvulus and risk of mortality in malawi. World J Surg 44(7):2087–2093. 10.1007/s00268-020-05440-2 32100066 10.1007/s00268-020-05440-2 PMC7272273

[21] Maya-Enero S et al (2022) Distinguishing outcomes of neonatal intestinal volvulus: review of our experience over the last 20 years. Acta Paediatr 111(2):284–290. 10.1111/apa.16167 34704280 10.1111/apa.16167

